# On the Lancet

**Published:** 1881-01

**Authors:** T. C. Osborne

**Affiliations:** Bonner, La.


ARTICLE III.

### ON THE LANCET.

BY PROF. T. C. OSBORNE, M. D.

BONNER, LA., *November 10th, 1880.*

*Prof. Harvey L. Byrd, M. D.*

MY DEAR DOCTOR :—It is very commendable in you calling attention to "Blood Letting in Disease," and asking an expression of opinion regarding its employment as a remedial agent.

Lukewarmness in the use of the lancet can never be charged to my account, during the earlier years of my professional career. On the contrary, my demands for it were nearly incessant, finding a necessity for it in almost all classes of disease, and deeming the remedy serviceable in many cases, which, perhaps, heads more cautious than mine would have hesitated in trusting to its powerful energies. In acute as well as chronic cases of intermittent fever, it was a frequent habit with me to bleed, in some cases, just before the expected paroxysm ; in others, during the chill ; and in yet others, just after the exascerbation had commenced. In hemoptisis, in menorrhagia, and other forms of hemorrhage. In dyspepsia, dysentery, and phthisis pulmonalis; and, finally, I have to confess to venesection in quite a number of cases of obstetrics, for the purpose of regulating and concentrating uterine contracting, as well in many cases, as to relax rigidity of the soft parts. And to sustain me in this practice, I had the teachings of Dewes, Rush, Drake and a great number of other professional lights in their day; and, after all, it is comforting to remember that in no instance have I ever had occasion to regret it; whilst, on the other hand, many things of regret have occurred to me for not bleeding, on account of the pressure of public opinion.

Until quite lately, advocates of the lancet have been reticent, but not convinced; and, I dare say, the time is coming when blood letting in disease will regain much, if not all the ascendency it once had as an active and powerful remedial agent. Diseases never change, they preserve their identity forever; but diathesis is more or less subservient to epidemic influences, and does not regain its sthenic character until after many consecutive years of general healthfulness. It is not too persevering, therefore, to assert that it is to this cause more than any other why the advocates of blood-letting have been silent; and why its opponents have been so imperious in its denunciation. If the healthy status of the past six years continues longer, it is altogether probable that the inflammatory diathesis will again assert itself, reversing the present order of practice, and call out the lancet from its hiding-place as a measure of profound necessity.

Admitting the probability that blood-letting was too often abused, and that many instances were known where possible injuries were sustained in susceptible organizations; it may be well answered that the same argument can, with equal force, be applied to all the agencies at our command, and that this cause alone could never have so emphatically retired the use of the lancet, had not so great a change come upon diathesis as was realized between the years 1835 and 1850. Those were remarkable times, and their ear marks have remained upon the population of these United States until but a short while back.

Away there in 1840, how well I remember those days of gloom and confusion in the ranks of the profession! It was figuratively as if we had collided with a chilling glacier during the existence of interminable fog, in which condition it was necessary to throw overboard our stock in trade — the lancet, colomel and tartar-emetic — to keep the ship afloat until a beam of sunshine could enable us to get our proper bearings. In another figure, we had a hard battle to fight, and our forces were nearly demoralized by the frequent defeats we met at every turn. Our most heroic armamentarium was nearly valueless in our hands; and to make matters still worse in our ranks, Thomsonianism sprang into existence as from an ambush, routing us in the South "horse, foot and dragoons." The people adopted the mushroom and were clamorous over it: and no wonder, for the Steam Doctors were doing what we found ourselves unable to do — curing their patients!

The State of Alabama adopted, and, by legislative enactment, protected that system of quackery, on account of the success it met with in the treatment of the congestive fevers of that period. And, I frankly confess it, the Steamers met and unhorsed me many times, both in that State and in the State of Tennessee, before I became conscious that it was not these feeble opponents which did the work, but my own blindness, failing to recognize the asthenic diathesis which was overshadowing all classes of disease. In this view of it Thompson was nearer right than we were. And, Yankee like, he availed himself of the protection of the patent office, in thrusting his system upon the people: and, strange as the admission may appear, the steaming practice was like the one thing needed to clear away the fogginess which was so severe by threatening to engulf us in total darkness.

In the great pandemic wave of 1839, 1840 and 1841—of malarial fevers—more than all others combined, is to be traced the decline of the practice of blood-letting; the silence of the advocates of the lancet, tartar-emetic and calomel; and the outspoken denunciations of the opponents of this form of practice.

That rare old medical philosopher, Dr. Daniel Drake, then of Louisville, Kentucky, made a tour of observation during these memorable times, stopping patiently at all important points along his route, taking notes for his forthcoming book on "The Principal Diseases of the Valley of North America," and to his timely suggestions made through the pages of the "Western Journal of Medicine and Surgery," we were indebted for much valuable information on the subject of malaria in its effects as lingering impressions upon diathesis. The idea was, however, beginning to obtain in the profession that we had asthenic to contend with, and that the old system of depletive remedies would have to be abandoned, and a conservative treatment adopted in its stead.

No part of the interval between that great malarial epidemic and the next one, which occurred in 1866, 1867 and 1868, was entirely a period of freedom from epidemic influences. In 1848 there was a wave of cholera along the blue limestone belt, and a tipho-malarial fever epidemic south of it. In 1856 there was a wide-spread wave of dysentery ; and in 1858 we had to contend with an epidemic form of diphtheria, which oscillated through several years until the time of the fratricidal war, which seemed to arrest its progress, and allow a breathing spell from the ravages of disease at home· The wave of 1867—for that was the period of its greatest intensity—was less violent than that of 1840, and did not impose afterwards upon diathesis in anything like the same manner, nor to the same extent; and the consequence is, the impressions made by it have, in a great measure passed away, leaving a tendency to a return of the sthenic status which existed prior to the latter-mentioned period, 1840.

Having passed through these two pandemic waves of malarial diseases, and having carefully observed the phenomena accompanying them, I feel somewhat warranted in submitting in this place a few propositions as data for comparative observations on the part of others.

In the first place, these great malarial epidemics occupy and linger through three years time, beginning with intermittent in the spring, remittent in the summer, congestive and continued fever in the autumn, and prenumonias in the winter months; culminating in intensity in the autumn of the second year, and, after the third year, followed by a train of nervous disorders, such as neuralgias, chorea and hysterical phenomena.

2. Between their returns we have other kinds of epidemics, which are traceable to the impressions left upon a susceptible diathesis.

3. They return with something like regularity, moving from the South, as was witnessed in the wave of 1820, 1821 and 1822; in 1839, 1840 and 1841; and, finally, in 1866, 1867 and 1868—nineteen years between the first and second; and twenty-seven years between the second and third periods.

4. Epidemic cholera arises in the East and moves towards the West, observing certain limits in its oscillation, but never has, so far, appeared at the same time with the malarial fever epidemics, and consequently these two great scourges of the human family do not cross and conflict in any portion of the world.

And now, my dear Doctor, having presumed so far upon your patience, I beg the liberty of submitting my paper to your better judgment, and subscribe myself with much respect.

Yours cordially, T. C. OSBORNE, M. D.

